# Extracranial Medulloblastoma With Myogenic Differentiation: Report of a Rare Event

**DOI:** 10.7759/cureus.48301

**Published:** 2023-11-05

**Authors:** Afnan Elsayed, Hosam Alardati, Jaudah Al-Maghrabi, Abdelrazak Meliti

**Affiliations:** 1 Pathology and Laboratory Medicine, King Faisal Specialist Hospital and Research Centre, Jeddah, SAU; 2 Department of Pathology, Faculty of Medicine, King Abdulaziz University, Jeddah, Saudi Arabia, Jeddah, SAU; 3 Pathology and Laboratory Medicine, Alfaisal University, Riyadh, SAU

**Keywords:** extracranial metastasis, myogenic differentiation, medulloblastoma, malignant tumor, lymph node metastasis

## Abstract

Medulloblastoma accounts for nearly 10% of childhood primary central nervous system (CNS) malignancies. However, it is rare in adults. Extracranial metastasis is commonly documented to involve bones but rarely involves lymph nodes. Herein, we present an unusual case of primary CNS medulloblastoma in an adult patient with extracranial metastasis to a lymph node, which exhibits a myogenic differentiation. To the best of our knowledge, this is the fourth reported case of medulloblastoma in an adult with extracranial metastasis to the lymph node and the first reported case of extracranial metastatic medulloblastoma with myogenic differentiation that involves a lymph node.

## Introduction

Medulloblastoma is an aggressive embryonal tumor that accounts for nearly 10% of childhood primary central nervous system malignancy. It is the second most common central nervous system (CNS) malignant tumor in childhood. However, it is rare in adults [1]. Four molecularly defined subtypes of medulloblastoma are described in the World Health Organization (WHO) Blue Books, 5th edition (medulloblastoma, WNT-activated/medulloblastoma, SHH-activated and TP53-wildtype/medduloblastoma, SHH-activated and TP53-mutant/medulloblastoma, and non-WNT/non-SHH). Morphologically, four subtypes are described (classic, desmoplastic/nodular, meduloblastoma with extensive nodularity, and large cell/anaplastic medulloblastoma). The median patient age at diagnosis of medulloblastoma is nine years, with peaks in incidence at three and seven years of age. The annual childhood incidence is six cases per one million. Medulloblastoma occurs exclusively in the posterior fossa and has the potential for leptomeningeal spread. Surgical debulking is the main therapy in addition to radiation therapy and chemotherapy. In addition, other promising modalities, including immune checkpoint inhibitors, oncolytic viruses, chimeric antigen receptor (CAR) T-cell therapy, and natural killer (NK) cells in recurrent and refractory medulloblastoma [2]. Extracranial metastases carry a poor prognosis, most commonly involving bones and rarely lymph nodes [3]. The tumor cells are positive for synaptophysin and chromogranin. Interestingly, the tumor exhibits immunoreactivity to smooth muscle actin (SMA), muscle-specific actin (MSA), and desmin, a rare phenomenon in medulloblastoma.

## Case presentation

**Figure 1:**
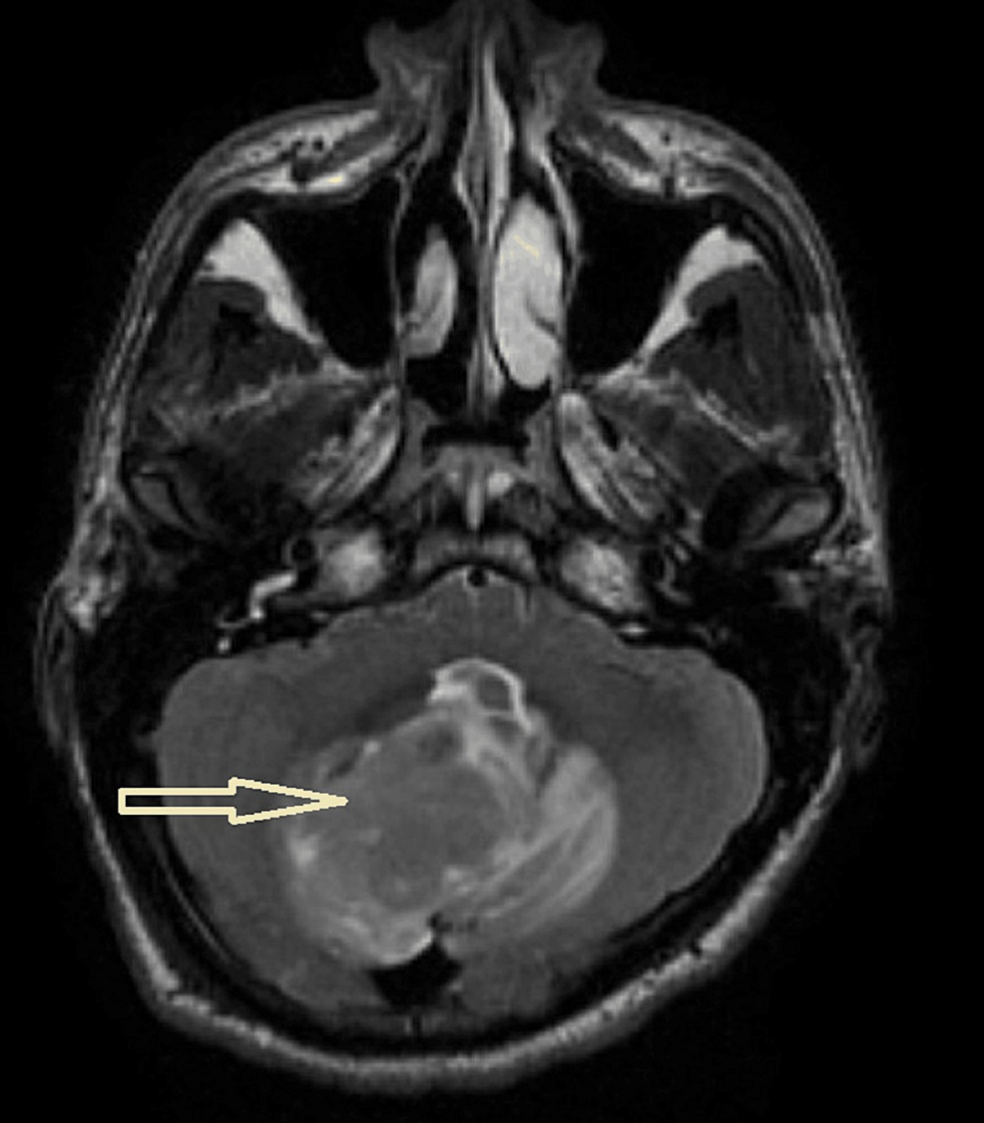
MRI of the brain MRI of the brain demonstrates a large intra-axial/infra-tentorial mass, mainly involving the right cerebellar hemisphere (arrow)

A 20-year-old male patient, medically free, presented with a history of headache and blurring of vision for several weeks. Cranial magnetic resonance imaging (MRI) revealed a large intra-axial posterior fossa mass (4.3 x 4.1 x 3.9 cm), causing significant compression on the fourth ventricle with cerebellar tonsillar herniation at the foramen magnum (Fig. 1).

The surgical approach to posterior fossa medulloblastoma includes a midline suboccipital craniotomy. The surgeons were able to achieve gross total resection of the tumor, confirmed by post-operative MRI. The post-operative cerebrospinal fluid (CSF) examination was negative. The histopathological diagnosis was medulloblastoma, a desmoplastic/nodular variant. That was followed by radiotherapy (36 Gy in 20 fractions to the craniospinal axis, with a posterior fossa boost of 18 Gy in 10 fractions). Subsequent MRI scans at routine follow-up appointments revealed no evidence of recurrence.

**Figure 2:**
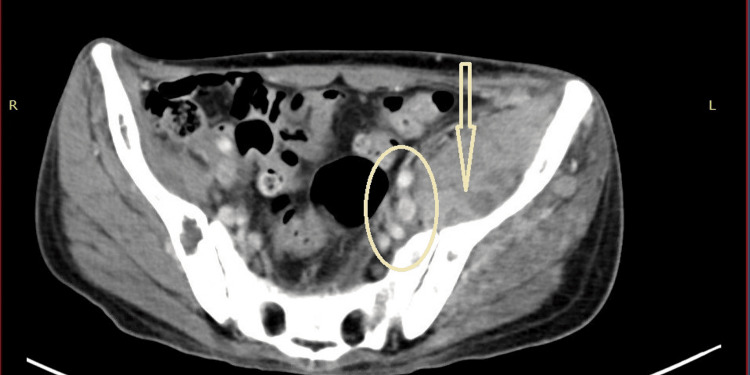
CT scan abdomen The arrow indicates a large soft tissue mass involving the left iliac fossa; the circle indicates left pelvic lymphadenopathy.

**Figure 3:**
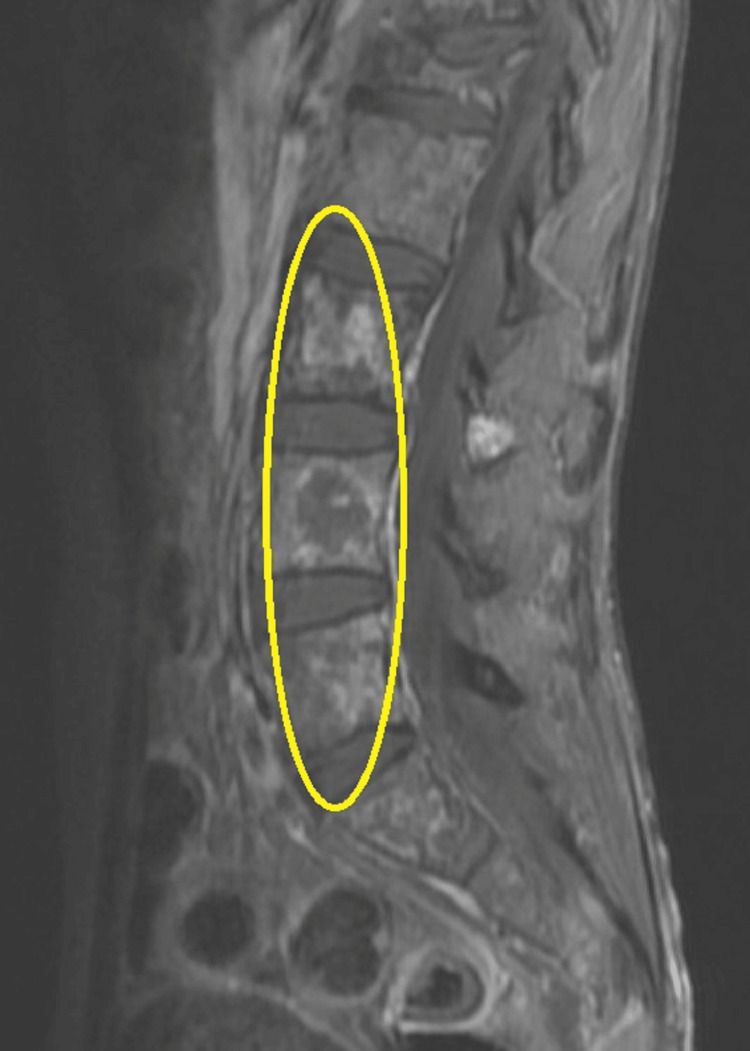
MRI of the abdomen/sagittal section of the lumbosacral region MRI of the abdomen/sagittal section demonstrating destructive bony metastasis involving the lower lumbar vertebrae (L3-L5) (yellow circle).

Two years later, the patient presented with left inguinal swelling and limited movement on his left lower limb. Abdominal CT scan with contrast revealed extensive metastatic lymphadenopathy and a large metastatic soft tissue mass predominantly involving the left side of the pelvis (Fig. 2), and an MRI of the abdomen demonstrates extensive destructive bony lesions of the lumbosacral vertebrae (Fig. 3).

**Figure 4:**
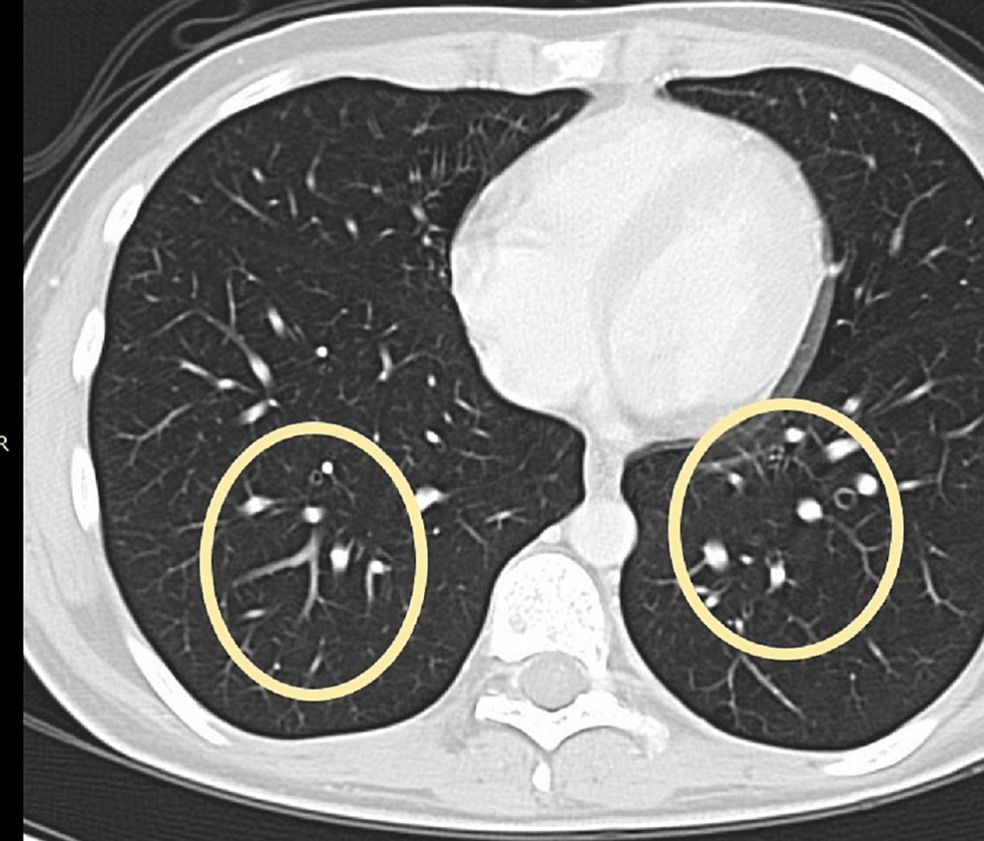
CT scan of the chest demonstrating multiple bilateral metastatic nodules (circles).

CT chest with contrast revealed multiple bilateral nodular lung opacities (Fig. 4).

**Figure 5:**
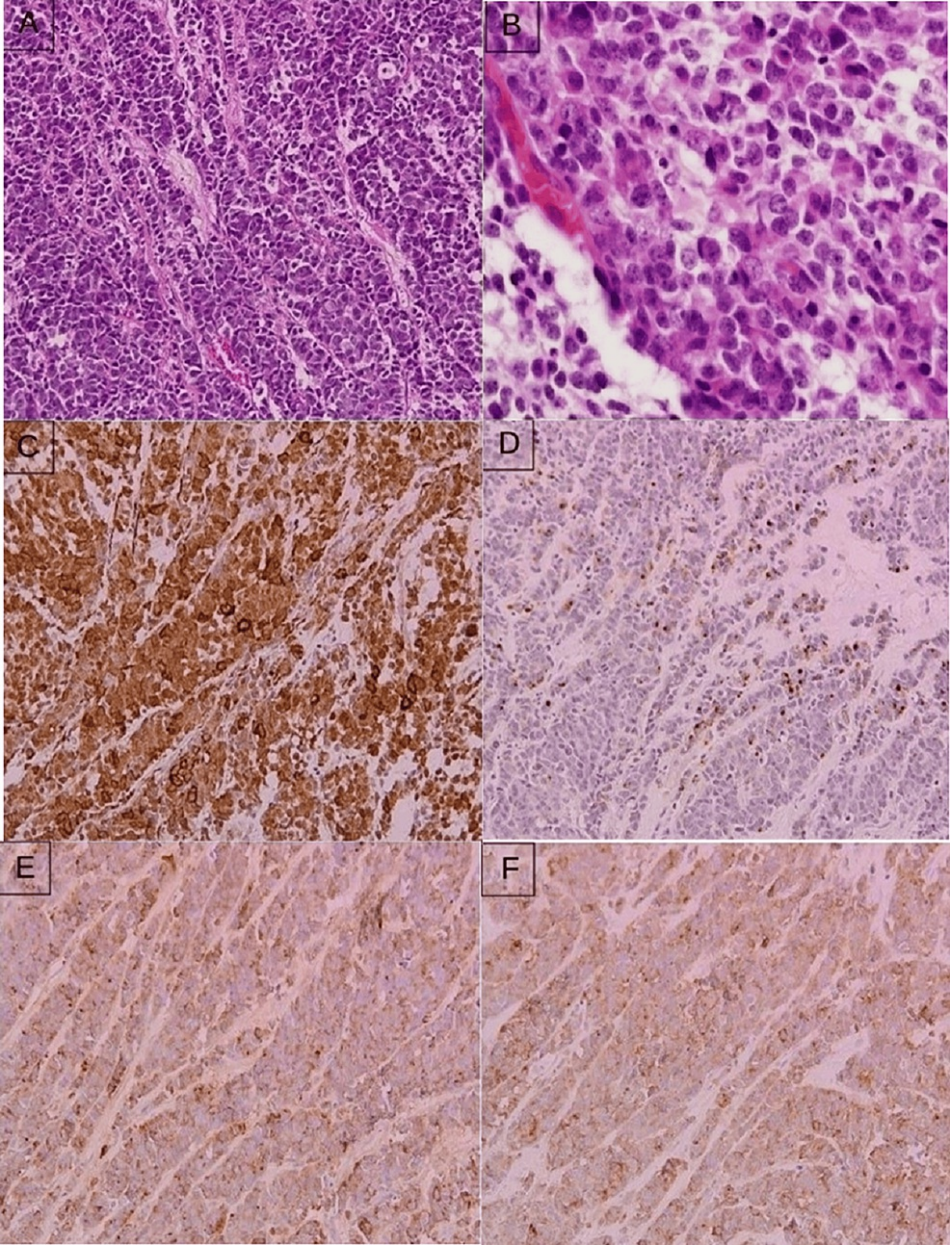
Histopathology sections in hematoxylin and eosin stain (H&E) and immunohistochemistry studies from the lymph node (A) Multinodular growth pattern, which is composed of small- to medium-sized cells with hyperchromatic nuclei, scant cytoplasm, and focal molding (hematoxylin and eosin (H&E): 20x). (B) Tumor shows a proportion of cells with eccentric nuclei and bright eosinophilic cytoplasm (H&E: 100x). (C) Tumor cells are strongly and diffusely positive for smooth muscle actin (SMA) (H&E: 20x). (D) Focally positive for desmin (H&E: 20x). (E&F) Tumor cells are positive for chromogranin and synaptophysin, respectively (H&E: 20x).

The subsequent MRI of the brain revealed no intracranial recurrence. The patient underwent excision of the left inguinal lymph node. Pathological examination and the immunohistochemistry studies of the lymph node revealed a poorly differentiated small round blue cell neoplasm with a multinodular and diffuse growth pattern, which was composed of small- to medium-sized cells with hyperchromatic nuclei, scant cytoplasm, and focal molding (Fig. 5A). Frequent mitotic figures were observed. The tumor showed a proportion of cells exhibiting eccentric nuclei and bright eosinophilic cytoplasm suggestive of myogenic differentiation (Fig. 5B). The cells were positive for smooth muscle actin (SMA) (Fig. 5C), patchy staining for muscle-specific actin (MSA), and focally positive for desmin (Fig. 5D). Chromogranin (Fig. 5E) and synaptophysin were positive throughout the tumor cells (Fig. 5F)

Meanwhile, Myo D1, myogenin, CD99, FLI1, epithelial membrane antigen (EMA), S100, Pankeratin, CD45, CD20, and CD3 were negative. Integrase interactor1 (INI1) showed retained nuclear positivity. The final histopathological diagnosis was a malignant, small, round blue cell tumor consistent with extracranial metastatic medulloblastoma with myogenic differentiation. A retrospective immunohistochemical stain of the primary CNS medulloblastoma was performed. The neoplastic cells were positive for SMA and MSA while negative for desmin. 

Later, the patient received palliative chemotherapy and radiotherapy with prompt resolution of symptoms. A subsequent CT scan revealed an interval improvement of the previously noted metastatic lymphadenopathy. On follow-up, the patient's condition deteriorated, and he developed signs and symptoms of acute kidney injury, bilateral hydro-nephrosis, and thrombocytopenia secondary to chemotherapy. He expired 10 months after the diagnosis of metastasis.

## Discussion

Medulloblastoma was first named after Baily and Cushing in 1952 and was described as an aggressive, highly malignant tumor of the midline cerebellum [4]. It is the most common CNS tumor in childhood and is rare in adulthood. They account for 40% of all posterior fossa tumors in children and represent only 0.5-1% of all adult intracranial tumors [5].

Extracranial metastases from medulloblastoma most commonly involve the bone, accounting for 88% of all cases, especially the axial bone, including the vertebrae and ribs. By contrast, metastases pattern from the primary CNS tumors, especially gliomas, where the lung is the most common site of the extracranial metastases and bones are the less common. Lymph node metastases from medulloblastoma are exceptionally rare and usually are associated with or proceeded by bone metastases [6]. The majority of cases occur sporadically, but some have been associated with hereditary conditions, such as Gorlin syndrome, Rubinstein-Taybi syndrome, ataxia-telangiectasia, Li-Fraumeni syndrome, tuberous sclerosis, and neurofibromatosis. The four major histological variants are classic medulloblastoma, desmoplastic/nodular medulloblastoma, medulloblastoma with extensive nodularity, and large cell/anaplastic medulloblastoma [7]. In addition, at least four molecular subgroups of medulloblastoma have been recognized: WNT, sonic hedgehog (SHH), Group 3, and Group 4 [8]. The histogenesis of medulloblastoma with myogenic differentiation is controversial; some suggest that these tumors originate from the pluripotent stem cells of the fetal cerebellar meninges [9]. Others propose that myoblastic cells originate from the neoplastic transformation of the endothelial cells as the muscle fibers are found near blood vessels [10].

An extensive search in the English literature revealed three reported cases of adult medulloblastoma with extracranial metastasis to the lymph node. Frankel et al. [11] reported a case of an adult medulloblastoma with extracranial metastases to the left cervical lymph node, left supraclavicular lymph node, and right iliac lymph node, respectively.

Medulloblastoma, with myogenic differentiation, also known as medullomyoblastoma, is an exceptionally rare variant of medulloblastoma, first coined by Marinesco and Goldstin in 1933 [12].

In the present case, the microscopic examination of the inguinal lymph node revealed a poorly differentiated small round blue cell neoplasm with foci of myogenic differentiation in the form of eccentric nuclei and bright eosinophilic cytoplasm, which were positive for SMA, MSA, and desmin (focally). A retrospective immunohistochemical stain of the primary CNS medulloblastoma was performed, which showed reactivity to SMA and MSA. Although the earlier WHO, in 2000, recognized medulloblastoma with myogenic differentiation as a separate variant of medulloblastoma, they are no longer considered distinct entities due to their lack of specific clinical or genetic features, but recently regarded as a pattern of differentiation that can occur in any of the four WHO histological variants [13].

A potential diagnostic pitfall in such a case includes the presence of myogenic differentiation and the rarity of lymph node metastases from medulloblastoma. The primary differential diagnosis of medulloblastoma with myogenic differentiation includes rhabdomyosarcoma and malignant rhabdoid tumor. Retained nuclear immunopositivity for INI1 ruled out rhabdoid tumor, and the lack of immunohistochemical reactivity to myogenin, MyoD1, focal expression of desmin, and positivity for synaptophysin and chromogranin excluded the diagnosis of rhabdomyosarcoma. Other differential diagnosis includes lymphoma, Ewing's sarcoma/PNET, and poorly differentiated carcinoma. CD45, CD20, CD3, FLI1, CD99, EMA, and Pankeratin immunohistochemical stains were negative, which exclude the other differential diagnosis. The prognosis of medulloblastoma with myogenic differentiation depends on the associated WHO histological variant molecular subgroup and has the same treatment.

Extracranial metastases carry a poor prognosis. Rochkind et al. [14] concluded that the mean survival (after diagnosing extracranial metastasis) in adults and children was 10 and five months, respectively.

## Conclusions

Medulloblastoma with myogenic differentiation is a rare variant of medulloblastoma that can be associated with any of the four WHO histological variants. Herein, we report a unique case of medulloblastoma with myogenic differentiation and extracranial metastasis to an inguinal lymph node in an adult patient. Pathologists should always consider metastasis in the differential diagnosis of lymphadenopathy in a patient with a history of primary brain tumors.
